# Intestinal Anti-Inflammatory Activity of Lentinan: Influence on IL-8 and TNFR1 Expression in Intestinal Epithelial Cells

**DOI:** 10.1371/journal.pone.0062441

**Published:** 2013-04-22

**Authors:** Yosuke Nishitani, Ling Zhang, Masaru Yoshida, Takeshi Azuma, Kazuki Kanazawa, Takashi Hashimoto, Masashi Mizuno

**Affiliations:** 1 Team of Health Bioscience, Organization of Advanced Science and Technology, Kobe University, Kobe, Japan; 2 Department of Agrobioscience, Graduate School of Agricultural Science, Kobe University, Kobe, Japan; 3 Gastroenterology Division, Department of Internal Medicine, Graduate School of Medicine, Kobe University, Kobe, Japan; 4 The Integrated Center for Mass Spectrometry, Graduate School of Medicine, Kobe University, Kobe, Japan; Institut Pasteur de Lille, France

## Abstract

Inflammatory bowel disease (IBD) is characterized by chronic inflammation of the gastrointestinal tract. It is unknown whether β-1,3;1,6-glucan can induce immune suppressive effects. Here, we study intestinal anti-inflammatory activity of *Lentinula edodes*-derived β-1,3;1,6-glucan, which is known as lentinan. Dextran sulfate sodium (DSS)-induced colitis mice were used to elucidate effects of lentinan *in vivo*. In the cellular level assessment, lentinan was added into a co-culture model consisting of intestinal epithelial Caco-2 cells and LPS-stimulated macrophage RAW264.7 cells. Ligated intestinal loop assay was performed for assessing effects of lentinan on intestinal epithelial cells (IECs) *in vivo*. Oral administration of lentinan (100 µg/mouse) significantly ameliorated DSS-induced colitis in body weight loss, shortening of colon lengths, histological score, and inflammatory cytokine mRNA expression in inflamed tissues. Lentinan reduced interleukin (IL)-8 mRNA expression and nuclear factor (NF)-κB activation in Caco-2 cells without decreasing of tumor necrosis factor (TNF)-α production from RAW264.7 cells. Flow cytometric analysis revealed that surface levels of TNF receptor (TNFR) 1 were decreased by lentinan treatment. A clathrin-mediated endocytosis inhibitor, monodansylcadaverine, canceled lentinan inhibition of IL-8 mRNA expression. Moreover, lentinan inhibited TNFR1 expression in Caco-2 cells in both protein and mRNA level. Lentinan also inhibited TNFR1 mRNA expression in mouse IECs. These results suggest that lentinan exhibits intestinal anti-inflammatory activity through inhibition of IL-8 mRNA expression associated with the inhibition of NF-κB activation which is triggered by TNFR1 endocytosis and lowering of their expression in IECs. Lentinan may be effective for the treatment of gut inflammation including IBD.

## Introduction

The gastrointestinal tract of higher organisms is lined with a single layer of IECs. This physical barrier separates subepithelial mucosal immune cells such as lymphocytes, macrophages, and dendritic cells from a variety of antigenic substances present within the intestinal lumen including bacteria and food antigens [1], [2]. The integrity of the epithelial barrier is essential for the maintenance of host homeostasis, as it prevents dysregulated uptake of luminal antigens. The incidence and prevalence of Crohn’s disease and ulcerative colitis, collectively referred to as inflammatory bowel disease (IBD), have been increasing worldwide [3]. IBD is characterized by chronic inflammation of the gastrointestinal tract. The clinical features of Crohn’s disease include diarrhea, pain, narrowing of the gut lumen leading to strictures and bowel obstruction, abscess formation, and fistulization of the skin and internal organs. Meanwhile, the clinical features of ulcerative colitis include severe diarrhea, blood loss, and progressive loss of peristaltic function [4]. While their precise etiology still remains unknown, understanding of the pathophysiology of IBD has advanced, and the typical features of these diseases have been shown in various studies, especially for intestinal immune cells and IECs of IBD patients. It has been reported that IECs, macrophages, and T cells secrete large amounts of chemokines such as IL-8 and pro-inflammatory cytokines including TNF-α, IL-6, IL-12, IL-17, IL-23, and interferon (IFN)-γ in the inflamed intestines of IBD patients [5]. IL-8 is a member of the C-X-C chemokines and is secreted excessively by a variety of cells at the site of inflammation, such as IECs, in IBD [6]. IL-8 causes excessive recruitment and transmigration of neutrophils into inflamed tissues followed by injury to the epithelia [7]. Lamina propria mononuclear cells (LPMCs) from patients with Crohn’s disease spontaneously secreted TNF-α [8]. It is well-known that the secretion of inflammatory cytokines like IL-8 and TNF-α may be an important part of the immune response, and the dysregulation of these cytokines is implicated in the pathogenesis of IBD [9], [10].

It has been reported that β-glucans derived from fungi and yeast possess immune modulating properties [11]. β-Glucans can enhance the functional activity of macrophages and activate the antimicrobial activity of mononuclear cells and neutrophils [12]–[14]. β-1,3-Glucans are major structural components of fungal cell walls which induce macrophage activation in mammals. Among the various β-1,3-glucans, lentinan possesses a structure composed of a backbone of β-1,3-linked glucose residues with side chains of β-1,6-glucose residues [15] and is an antitumor polysaccharide produced by *Lentinula edodes* [16]. Lentinan increases peritoneal macrophage cytotoxicity against metastatic tumors [17]. Lentinan can also activate the normal and alternative pathways of the complement system by splitting C3 into C3a and C3b, thereby enhancing macrophage activation [17]. Although a number of studies for the fascinating effect of β-1,3-glucans on the responsiveness or function of immune cells have been performed, their immune suppressive effects such as intestinal anti-inflammatory properties have not been studied sufficiently.

In our previous study, we established a gut inflammation *in vitro* model which is composed of intestinal epithelial Caco-2 cells and macrophage RAW264.7 cells [18]. When RAW264.7 cells were stimulated with lipopolysaccharide (LPS), IL-8 and TNF-α secretion increased. This gut inflammation model was used to search for anti-inflammatory factors that act against intestinal inflammation [18]. In the present study, we focused on the suppressive effect of lentinan on gut inflammation using an *in vivo* and an *in vitro* model and further examined its inhibitory mechanism.

## Materials and Methods

### Experimental Animals

#### Ethics statement

The care and use of the animals and experimental protocol were approved by the Guidelines for the Care and Use of Experimental Animals, of Rokkodai Campus, Kobe University, and were approved by the Animal Experiment Ethnics Committee of Kobe University (Permission number: 22-05-06).

Female, 6-week-old C57BL/6CrSlc mice were purchased from SLC (Shizuoka, Japan). Mice were housed in an air-conditioned animal room at 23±2°C with a 12-h light/dark cycle, and acclimated for 7 days. Mice were fed with a laboratory diet (Nihon Nosan, Yokohama, Japan) and water ad libitum.

### Reagents

Dulbecco’s Modified Eagle Medium (DMEM) mixed with glutamine containing 1.0 g/l glucose, LPS from *E. coli* O127, and recombinant murine TNF-α (rmTNF-α) were purchased from Wako Pure Chemical Industries (Osaka, Japan). MEM (Eagle’s Minimum Essential Medium) was purchased from Nissui Pharmaceutical (Tokyo, Japan). RPMI 1640 medium and MEM non-essential amino acids (NEAA) were purchased from Gibco BRL (Grand Island, NY). DMEM mixed with glutamine containing 4.5 g/l glucose, budesonide, cytochalasin D, and monodansylcadaverine were obtained from Sigma (St Louis, MO). Fetal bovine serum (FBS) was purchased from Biological Industries (Beit, Israel). Anti-human β-actin mouse monoclonal antibody (Ab) was purchased from Calbiochem (Darmstadt, Germany). Anti-human nuclear factor (NF)-κB p65 rabbit monoclonal antibody (Ab) and anti-human histone h1 mouse monoclonal Ab were obtained from Santa Cruz Biotechnology (Delaware Avenue, CA). Anti-human TNFR1 mouse monoclonal Ab was obtained from R&D Systems (Minneapolis, MN). Lentinan from *Lentinula edodes*, a dietary β-1,3;1,6-glucan used in this study, was gifted from Ajinomoto (Tokyo, Japan). Anti-lentinan rabbit polyclonal Ab has been reported in our previous study [19]. Other chemicals and reagents were ordinary commercial and guaranteed products.

### Induction of DSS Colitis

Colitis was induced in 7-week-old mice by administration of 2% (w/v) DSS (molecular weight; 36,000–50,000) to drinking water for 7 days. Lentinan (50, 100, and 200 µg per mouse) or vehicle was administered daily via intragastric administration, starting 7 days before DSS treatment and continuing until sacrifice. Doses of lentinan were chosen according to various reasons, such as the estimated human intake of mushroom [20], the pharmaceutical dose of lentinan via i.p. or i.v. injection for gastric cancer treatment [21], as well as our trial test. Mice were killed on day 10 (initial DSS treatment as day 1). For histological examination, the degree of inflammation and epithelial damage on hematoxylin and eosin (HE)-stained sections (8 µm) of distal colon was graded according to the method of Hudert *et al*. [22].

### Cell Culture

Cells from the human intestinal epithelial cell line Caco-2, obtained from American Type culture Collection (ATCC) (Manassas, VA, USA), were cultured in DMEM mixed with glutamine containing 4.5 g/l glucose, supplemented with 1% MEM-NEAA, 100 U/ml penicillin, 100 µg/ml streptomycin, and 10% decomplemented FBS (56°C, 30 min). Cell cultures were incubated in a humidified 5% CO_2_ incubator at 37°C. When the cells reached subconfluence, Caco-2 cells were recovered from the culture flask by trypsin digestion after being washed with phosphate buffered saline (PBS). Cells from the murine macrophage cell line RAW264.7, obtained from ATCC, were cultured in DMEM mixed with glutamine containing 1.0 g/l glucose and supplemented with 10% (v/v) decomplemented FBS (56°C, 30 min), 100 U/ml penicillin, and 100 µg/ml streptomycin. Cell cultures were performed in a humidified 5% CO_2_ incubator at 37°C and recovered in the same way as the Caco-2 cells described above. Cells from the murine fibrosarcoma cell line L929, obtained from ATCC, were cultured in MEM supplemented with 10% FBS, 2 mM L-glutamine, 100 U/ml penicillin, and 100 µg/ml streptomycin. Cell culturing was performed as for the Caco-2 cells described above.

### Co-culture System

A co-culture experiment was performed as described in a previous report [18]. For inhibition of receptor internalization, endocytosis inhibitors, including cytochalasin D (2 µM) and monodansylcadaverine (100 µM), were used. Before co-culturing with RAW264.7 cells, Caco-2 cells on transwell membrane were treated with an inhibitor or vehicle at 37°C for 30 min. After incubation, cells were washed three times with PBS, and then used in a co-culture model.

For assessing the effect of anti-lentinan Ab on lentinan activity, a rabbit polyclonal anti-lentinan Ab was used. The Ab was diluted with PBS at ratios of 1∶5 or 1∶100, and then mixed with lentinan solution and incubated on ice for 30 min. A rabbit polyclonal isotype control Ab was used as control at the same protein concentration as anti-lentinan Ab (dilution ratio of 1∶5).

### TNF-α Content Measurement

TNF-α contents were quantified with a cytotoxicity assay involving L929 cells (an actinomycin D-treated murine fibroblast cell line) using rmTNF-α as the standard as described by Kerékgyártó *et al*. [23].

### Western Blot Analysis

For Western blot analysis of NF-κB p65, nuclear protein was extracted as described by Zhang *et al*. [24]. For Western blot analysis of TNFR1, total protein was extracted with radioimmunoprecipitation (RIPA) buffer [1% Triton X-100, 0.5% deoxycholate, 0.1% sodium dodecyl sulfate (SDS), 2 mM PMSF, 2 mM ethylenediaminetetraacetic acid, and 2 mM orthovanadate] as described by Gaultier *et al*. [25]. The protein concentration was measured using the BCA™ Protein Assay Kit (Pierce, Rockford, IL). Equal amounts of the samples (5–30 µg) were mixed with sample buffer [125 mM Tris-HCl (pH 6.8), 4% SDS, 10% 2-mercaptoethanol, 0.2% bromophenol blue, and 20% glycerol] in a 1∶1 ratio, boiled for 10 min, and subjected to electrophoresis on 12.5% acrylamide gel. The electrophoresed proteins were transferred from the gel onto a polyvinylidene fluoride membrane. The membrane was blocked with TBST (Tris-buffered saline containing 0.1% Tween 20) containing 7% skimmed milk for 1 h. After being blocked, the membrane was incubated with primary Ab against histone h1 (1∶200), β-actin (1∶15,000), NF-κB p65 (1∶1,000), or TNFR1 (1∶1,000) for 1 h. Then, the membrane was incubated with secondary Ab for 1 h at room temperature. Peroxidase conjugated anti-mouse IgM goat monoclonal Ab (1∶1,500; Calbiochem) for β-actin primary Ab, peroxidase conjugated anti-rabbit IgG (H+L) goat Ab (1∶1,000; Wako) for NF-κB primary Ab, and peroxidase conjugated anti-mouse IgG (H+L) goat Ab (1∶10,000; Jacson Immuno Research Laboratories, West Grove, PA,) for TNFR1 and histone h1 primary Abs were used as secondary Ab. The ECL Plus™ Western blotting detection system (GE Healthcare) was used for blot detection according to the manufacturer’s protocol. The reagent was drained, and the membrane was exposed to a hyper film in the film cassette.

### RNA Isolation and Quantitative RT-PCR

Total RNA was isolated from cultured cells and colon tissues by using Sepasol RNA I super (Nacalai Tesque, Kyoto, Japan) and RNAqueous with Plant RNA Isolation Aid (Ambion, Austin, TX), respectively, according to the manufacturer’s protocol. The reverse transcription of the RNA for quantitative PCR was performed using High-Capacity cDNA Reverse Transcription Kits (Applied Biosystems, Foster City, CA). Quantitative PCR was performed by the 7500 Fast Real Time PCR system (Applied Biosystems) using TaqMan® Fast Universal PCR Master Mix (Applied Biosystems), and Gene Expression Assays for mouse pro-inflammatory cytokines (TNF-α, IL-6, IL-1β, and IFN-γ), MIP-2 and β-actin, and human GAPDH and IL-8 (Applied Biosystems), according to the manufacturer’s protocol. For all panels, the bars represent the ratio of target gene to endogenous gene expression, as determined by the relative quantification method (ΔΔCT) (mean ± S.E. of triplicate determination).

### Flow Cytometry

Cells were harvested by scraping into medium and pelleted by centrifugation at 4°C. The subsequent fixing and staining steps were performed at 4°C. Pellets were washed twice in 0.5% bovine serum albumin in PBS and incubated in FB (2% FBS in PBS) for 10 min on ice. Cells were pelleted and resuspended in 100 µl of FB with mouse anti-human TNFR1 (1∶50 dilution) or the isotype control and incubated for 30 min. Cells were washed twice in FB. Cells staining with anti-TNFR1 Ab were incubated with Alexa Fluor488-conjugated anti-mouse IgG (H+L) goat Ab (1∶400 dilution; Molecular Probes, Eugene, OR) for 30 min, followed by washing twice in FB, and fixed with 4% paraformaldehyde in PBS. FACS analysis was performed with a FACSVerse (BD Biosciences) and FACSuite software (BD Biosciences). For quantitative analysis, the geometric mean fluorescence intensities (gMFIs) of samples were plotted as percentages of the gMFI obtained from medium treated cells, with the following fomula: % surface TNFR1 = (lentinan gMFI – isotype control gMFI)/(medium gMFI – isotype control gMFI) ×100.

### Immunofluorescence Staining of NF-κB p65 in Caco-2 Cells

After the co-culture experiments, Caco-2 cells were fixed with methanol for 5 min, before being blocked for 30 min with 10% goat serum at room temperature. The cells were incubated with a 1∶50 dilution of anti-NF-κB p65 antibody at room temperature for 2 h. The cells were stained with Alexa Fluor488-conjugated anti-rabbit IgG (H+L) goat antibody as secondary antibodies (1∶400 dilution; Molecular Probes, Eugene, OR) for 1 h at room temperature. Nucleic acids were stained with propidium iodide (PI) (535/617) (1∶500; Molecular Probes). Images were acquired using a fluorescence microscope (BZ-9000; Keyence, Osaka, Japan). PI staining appeared red and NF-κB p65 staining green. A quantitative imaging assay was performed as described previously [26].

### Ligated Intestinal Loop Assay and Isolation of Epithelium

Mice were anaesthetized with avertin and kept warm with a 37°C warming pad during the assay. One hundred microliter of lentinan solution (1 mg/ml) or vehicle were injected into the separated two ligated intestinal loops which were located in ileum, respectively. After incubation for 1 h, the mice were killed and the ligated intestinal loops were excised from the intestine. IECs were isolated from mouse small intestine by modifying the method described by Hase *et al*. [27]. Briefly, intestinal loops were dissected from the mouse small intestine excluding Peyer’s patches (PPs) and soaked in Hank’s balanced salt solution (HBSS) containing 30 mM EDTA. After incubation on ice for 12 min, IECs were isolated by manipulation with a fine needle under stereomicroscopic monitoring. The isolated epithelial cell sheets were kept in ice-cold HBSS until RNA extraction.

### Statistical Analysis

Data are expressed as the mean ± SE. Statistical analysis was performed using the Student’s *t*-test. Statistical significance was defined as *P*<0.05. Statistical differences of colon lengths in DSS colitis mouse model were evaluated by analysis of variance (ANOVA) and Tukey-Kramer Multiple Comparisons Test to determine differences between groups. The results were considered significant when *P*<0.05.

See supplementary materials and methods for supporting information regarding lentinan content measurement (Text S1) and immunofluorescence staining of TNFR1 in Caco-2 cells (Text S2).

## Results

### Mouse Characteristics

**Figure 1:**
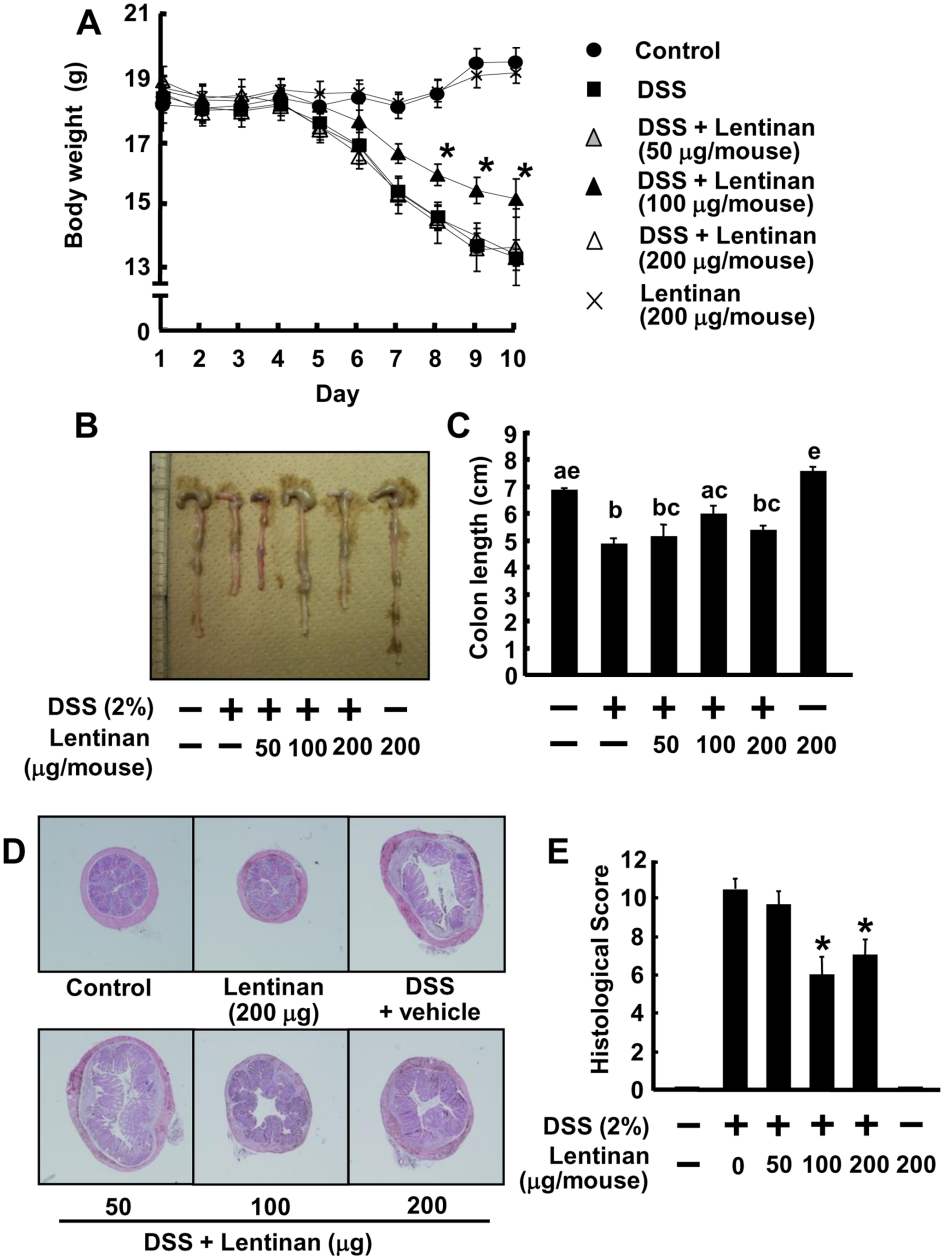
Lentinan treatment ameliorates DSS-induced colitis in mice. Oral administration of lentinan was started 7 days prior to DSS treatment. (A) Body weight changes of DSS-induced colitis mice with or without lentinan treatment according to the protocol described in materials and methods. Difference between 100 µg/mouse of lentinan-treated mice and non-treated DSS colitis mice was significant at *P*<0.05 (*). (B) Macroscopic appearance of a representative colon from wild-type (WT) and DSS-colitis mice with or without administration of lentinan. (C) Mean colon lengths were plotted. Items with different letter were significantly different (*P*<0.05). (D) Photograph (×40) of HE-stained paraffin sections of a representative colon from WT mice and DSS-colitis mice with or without administration of lentinan. (E) Histological scores of the colon sections of DSS-colitis mice with or without administration of lentinan. Significance compared to a DSS-treated group, *P<0.05. Values represent the means ± SE (*n* = 6). Experiments were repeated for three times.

To study the effect of lentinan on the innate immune response during colonic inflammation, an innate immune-mediated model of colitis induced by DSS was performed. DSS is a sulfated polysaccharide which can disrupt the mucosal epithelial barrier, thus exposing local macrophages to stimuli from the intestinal microflora [28]. Mice treated with DSS developed significant signs of IBD-like colitis, bloody diarrhea and wasting conditions with sluggish, weak movement, as well as a decrease in water and food uptake from day 4. In addition, a DSS-treated group decreased means of body weight in contrast to a vehicle-treated control group from day 5, and oral administration of 100 µg/mouse lentinan to DSS-induced colitis mice improved means of body weight (15.60±0.35 g on day 8, 15.08±0.44 g on day 9, and 14.82±0.62 g on day 10) which was significantly higher than that of DSS colitis mice (14.19±0.39 g, 13.33±0.33 g, and 12.86±0.29 g on corresponding day) (*P*<0.05) (Fig. 1A). Similar result was observed in the colon length. The colon length of DSS-treated mice with lentinan at the concentration of 100 µg/mouse (5.98±0.32 cm) was significantly longer than DSS-treated mice (4.87±0.22 cm) (Fig. 1B and 1C). These results suggest that oral administration of lentinan has inhibitory activity on body weight loss and shortening of the colon in DSS-induced colitis.

### Histological Inflammation Characteristics

Histological examination of intestinal tissue of mice after DSS colitis was performed at day 10. As shown in Fig. 1D and 1E, mice treated with DSS induced a significant increase of histological score compared with the vehicle-treated control mice, and the oral administration of 100 and 200 µg/mouse lentinan to DSS-induced colitis mice significantly inhibited the increase of histological score (*P*<0.05). These results suggest that oral administration of lentinan has an intestinal anti-inflammatory activity through alleviating severity of inflammation, inflammatory cell infiltration to colonic mucosa, as well as the degree and extent of epithelial damage.

### Cytokines and Chemokine Levels in the Colon

**Figure 2:**
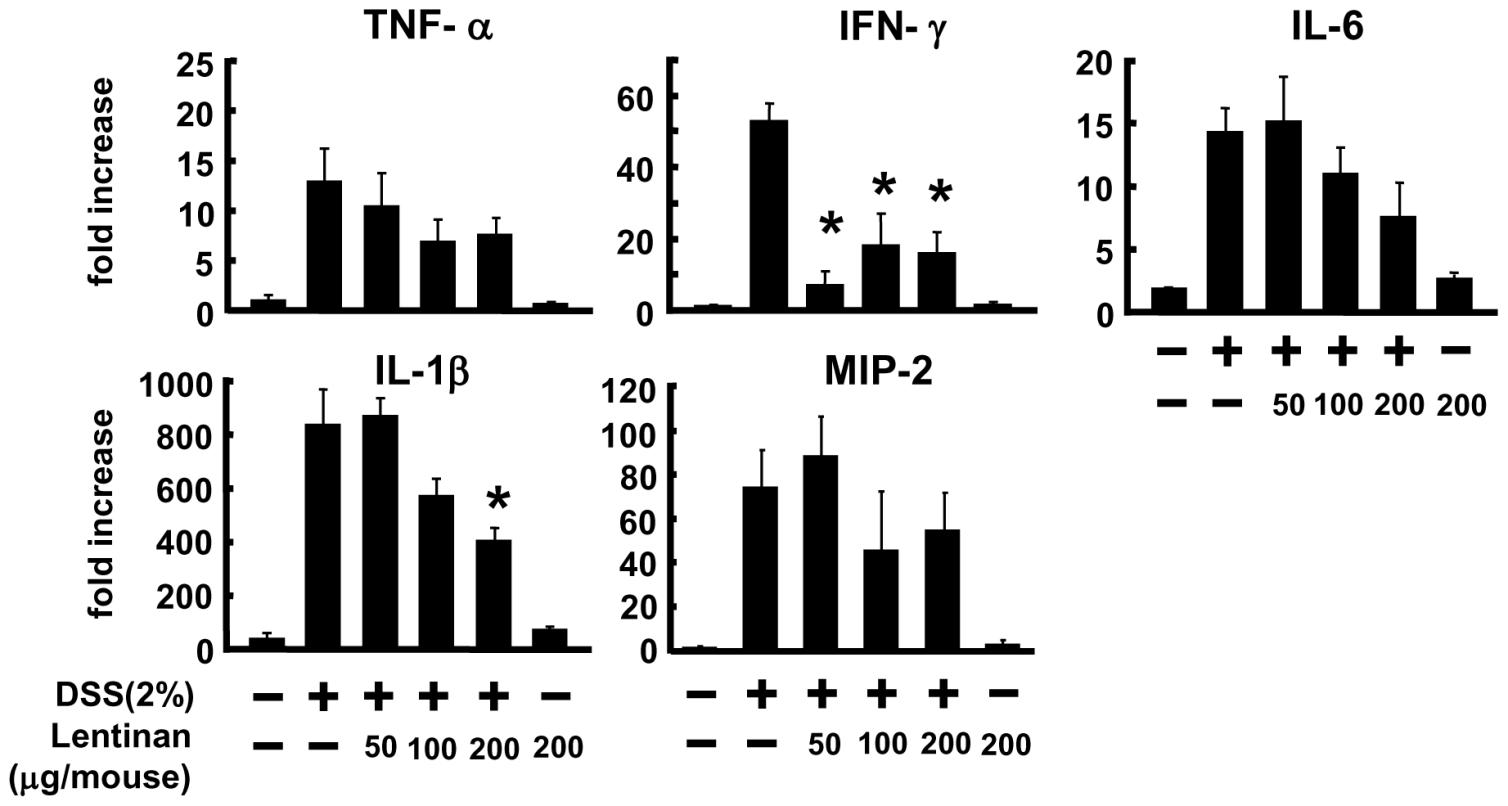
Lentinan improves inflammatory cytokines mRNA expression in colon tissues of DSS-induced colitis. Total RNA was extracted from the colon tissues of untreated and DSS-treated mice on day 10. TNF-α, IFN-γ, IL-6, IL-1β, and MIP-2 mRNA expression were measured by quantitative RT-PCR. Values represent the means ± SE (*n* = 6). Significance compared with a DSS-treated group, *P<0.05.

DSS-induced colitis model has been associated with increase in Th1 responses, therefore mRNA expression levels of several cytokines and chemokine in the whole colon tissue were analyzed by using real-time PCR. As shown in Fig. 2, compared with DSS-untreated mice, mRNA expression of pro-inflammatory cytokines such as TNF-α, IFN-γ, IL-6, and IL-1β increased dramatically in DSS-treated mice, and the treatment with lentinan improved the aberrant mRNA expression induced by DSS with significant reduction in Th1 type pro-inflammatory cytokine IFN-γ (100 and 200 µg/mouse of lentinan) and IL-1β (200 µg/mouse of lentinan) mRNA expression (*P*<0.05). These results suggest that oral administration of lentinan may exhibit anti-inflammatory activity via modulation of pro-inflammatory cytokines mRNA expression in the gut of DSS-induced colitis mice.

### Anti-inflammatory Effect of Lentinan on IL-8 mRNA Expression in the gut Inflammation Model of Caco-2 Cells and LPS-activated RAW264.7 Cells

In order to determine the inhibitory mechanism of lentinan, we used a previously established *in vitro* gut inflammation model [18]. In our previous study, treatment with lentinan (500 µg/ml) significantly reduced the IL-8 mRNA expression in Caco-2 cells (*P*<0.05) [29]. In the presence of rmTNF-α in the basolateral compartment, the IL-8 mRNA expression was increased (Fig. S1A). We could not detect TNF-α secretion in the apical compartment of this model (data not shown), and a previous study showed that anti-mouse TNF-α Ab treatment of the basolateral compartment in this model completely suppressed IL-8 mRNA expression [18]. In addition, when the gut inflammation model were performed with Caco-2 cells grown as monolayers on the underside of the transwell inserts (inverted position), addition of LPS into the lower chamber did not induce IL-8 mRNA expression in Caco-2 cells despite that TNF-α production from RAW264.7 cells was relatively normal (Fig. S1B and S1C). These results suggested that TNF-α in the basolateral compartment is essential for the up-regulation of the IL-8 mRNA expression of Caco-2 cells in this model. In our previous study, lentinan did not reduce the secretion of TNF-α when RAW264.7 cells were stimulated by LPS [29]. These results suggest that lentinan suppresses IL-8 mRNA expression in IECs without a reduction in TNF-α production. An ELISA inhibition technique using an anti-lentinan Ab was carried out to verify whether lentinan could penetrate a Caco-2 monolayer. Lentinan was not detected in the basolateral supernatant (data not shown), ascertaining that lentinan was not able to penetrate the Caco-2 monolayer in this model. These results indicate that the inhibitory activity of lentinan on IL-8 mRNA expression acted through the interaction between lentinan and Caco-2 cells, but not between lentinan and RAW264.7 cells in this model.

### Inhibitory Effect of Lentinan on NF-κB Translocation into the Nucleus of Caco-2 Cells

**Figure 3:**
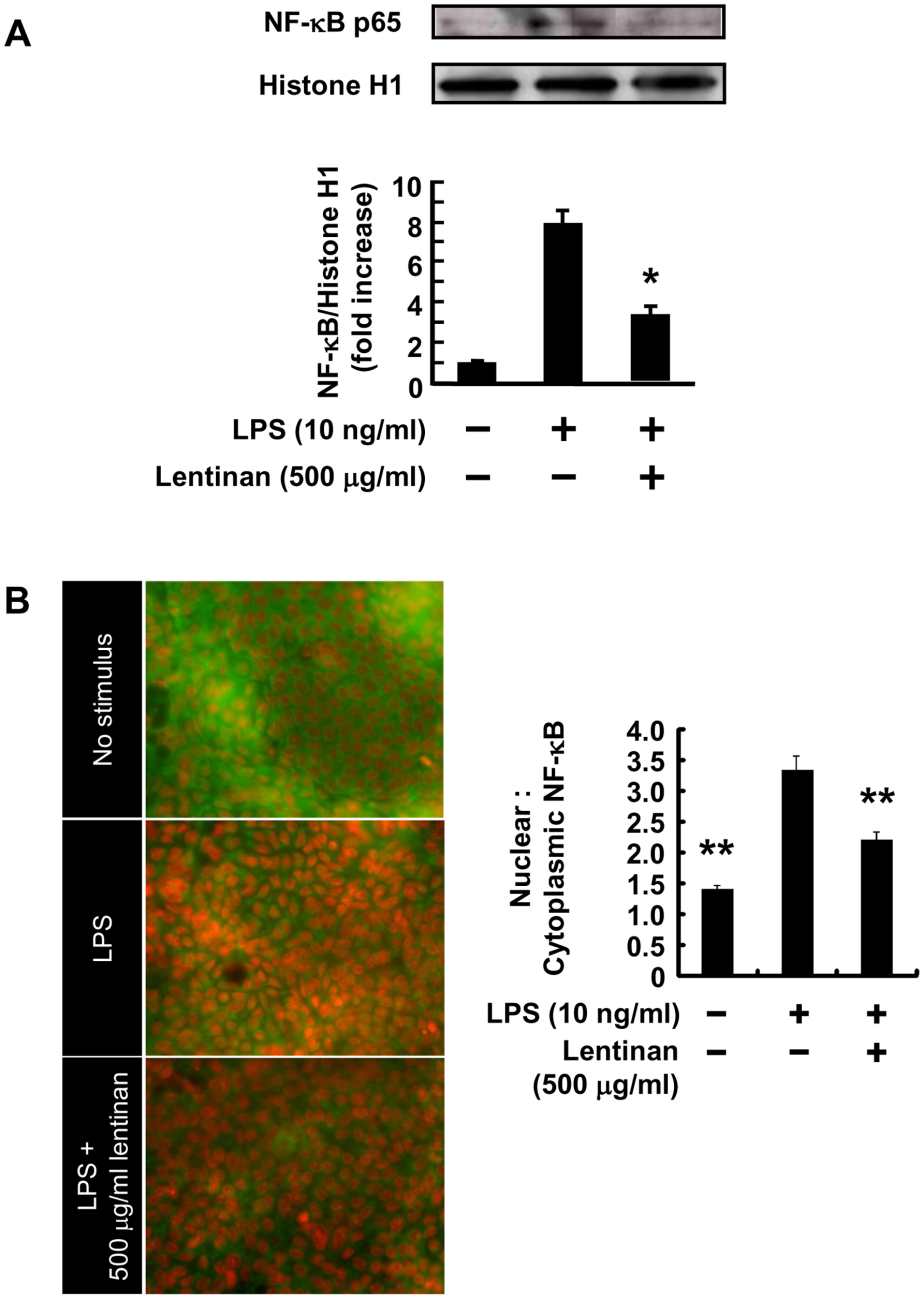
Lentinan inhibits NF-κB p65 nuclear translocation in Caco-2 cells. Lentinan (500 µg/ml) was added into the apical compartment of Caco-2/RAW264.7 co-culture model for 3 h. Subsequently, LPS was added to the basolateral compartment at a concentration of 10 ng/ml, followed by incubation for an additional 2 h. (A) Western blot analysis of the NF-κB p65 subunit was performed on nuclear extracts from Caco-2 cells. (B) Image analysis was performed according to the method described previously [26]. The values represent the means ± SE. Experiments were repeated for two times in triplicate. *P<0.05, **P<0.01 vs. LPS control.

The transcriptional activity of the human IL-8 promoter is known to be regulated by various transcriptional factors such as NF-κB [30]. In Caco-2/RAW264.7 gut inflammation model, an increase in IL-8 mRNA expression in Caco-2 cells was observed in association with TNF-α production from LPS-stimulated RAW264.7 cells [29]. Therefore, we examined the NF-κB p65 level in the nucleus of Caco-2 cells. As expected, the NF-κB p65 level in the nucleus peaked at 5 h incubation in association with TNF-α production (Fig. S2A and S2B). Subsequently, we assessed the effect of lentinan on NF-κB activation in Caco-2 cells. Western blot and immunofluorescence analysis showed that treatment with lentinan (500 µg/ml) significantly lessened the increase in NF-κB p65 level (Fig. 3A and 3B). These results suggest that the addition of lentinan into the apical compartment suppresses the NF-κB activation of Caco-2 cells in the gut inflammation model.

### Lentinan Reduces Surface Levels of TNFR1 in Caco-2 Cells

**Figure 4:**
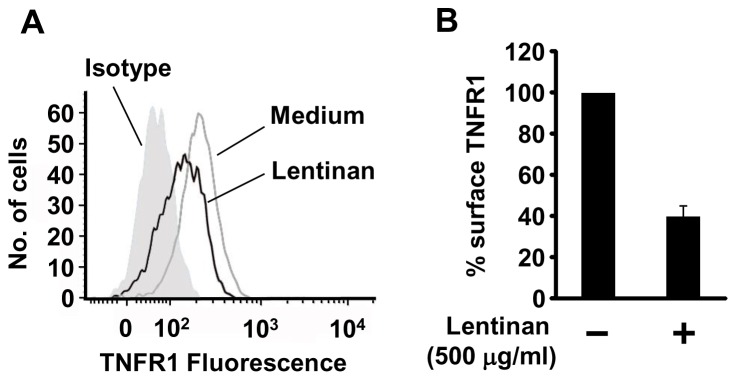
Lentinan suppresses cell surface levels of TNFR1 in Caco-2 cells. Lentinan (500 µg/ml) was added into the apical compartment of Caco-2/RAW264.7 co-culture model for 30 min. Subsequently, Caco-2 cells were harvested, fixed, and stained according to the method described in materials and methods. (A) Surface levels of TNFR1 were analyzed by flow cytometry. (B) The resulting gMFIs were plotted as percentages of the gMFI obtained from medium treated cells, with the following fomula: % surface TNFR1 = (lentinan gMFI – isotype control gMFI)/(medium gMFI – isotype control gMFI) ×100. The values represent the means ± SE. Experiments were repeated for three times in triplicate.

TNF-α transduces signals via specific receptors, and two TNFR isotypes, TNFR1 and TNFR2, are known. It has been reported that TNFR1 mediates TNF-α-induced NF-κB activation [31], [32], and TNFR1 signaling in IECs is crucial for the disease pathogenesis of inflammatory colitis in intestinal epithelial IκB kinase-γ (IKKγ) knockout mice [33]. We hypothesized that the ability of lentinan to regulate the TNFR1 level in Caco-2 cells accounts for the decrease in IL-8 mRNA expression, which is a downstream event of TNFR1-dependent cell signaling. To test this hypothesis, cell surface levels of TNFR1 in Caco-2 cells was determined by flow cytometric analysis. As expected, lentinan reduced surface TNFR1 in Caco-2 cells (Fig. 4A). The geometric mean fluorescence intensities (gMFI) of lentinan-treated cells were decreased approximately 60% compared with vehicle-treated cells (Fig. 4B). These results are consistent with the hypothesis that lentinan inhibits TNF-α-induced NF-κB activation by down-regulating cell surface TNFR1. The subsequent experiments are performed to study the mechanism involved in TNFR1 down-regulation by lentinan.

### Endocytosis Inhibitor Cancels Lentinan Inhibition of IL-8 mRNA Expression in Caco-2 Cells

**Figure 5:**
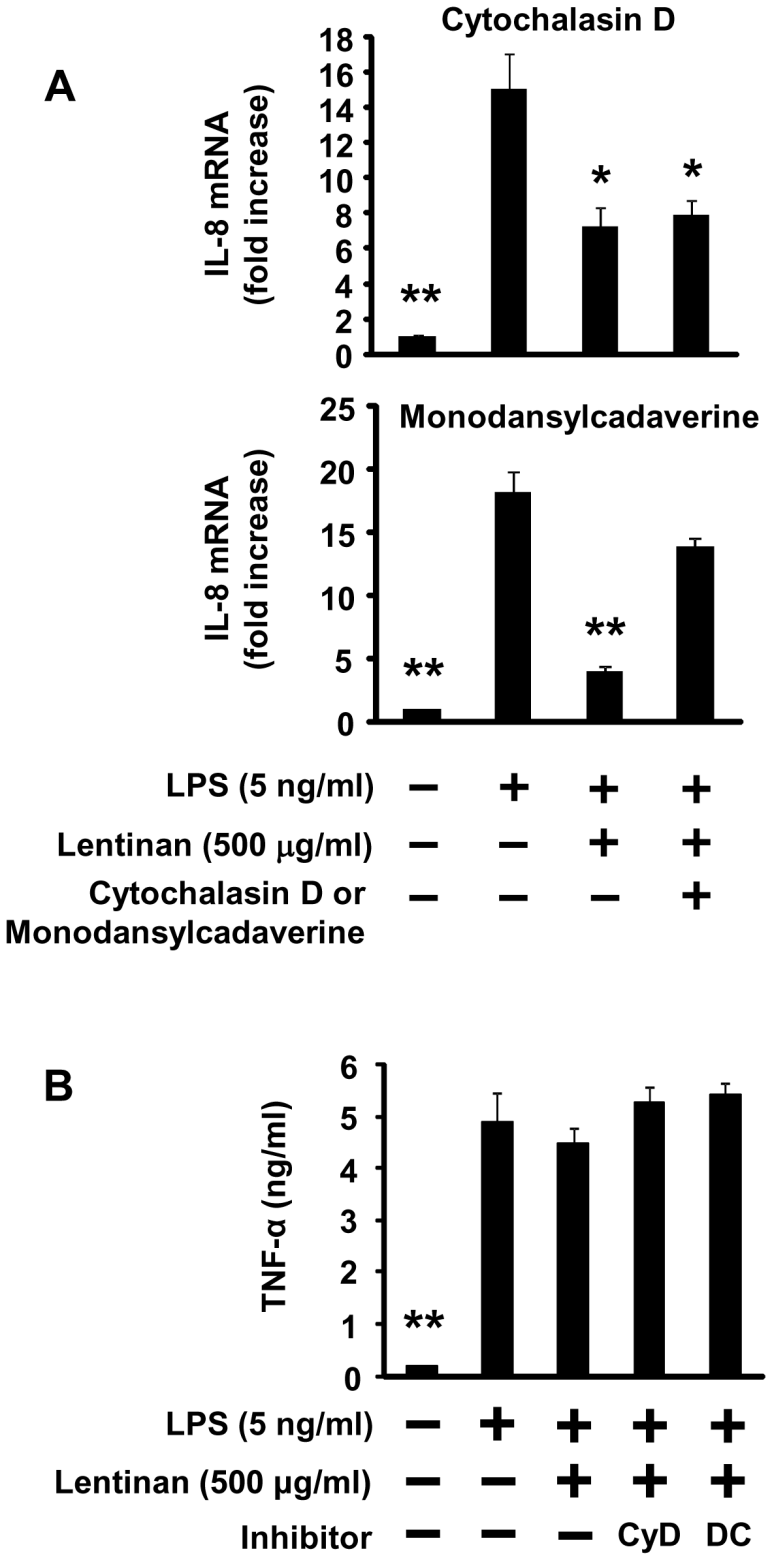
Effect of endocytosis inhibitors on lentinan inhibition of IL-8 mRNA expression in Caco-2 cells. Before co-culturing with RAW264.7 cells, Caco-2 cells on transwell membrane were treated with endocytosis inhibitors, cytochalasin D (2 µM) or monodansylcadaverine (100 µM), for 30 min. Caco-2 cells were washed three times with PBS, and then the cells were used in a co-culture model. Lentinan (500 µg/ml) was added into the apical compartment of a co-culture model for 3 h. Subsequently, LPS was added to the basolateral compartment at a concentration of 5 ng/ml, followed by incubation for an additional 3 h. IL-8 mRNA expression in Caco-2 cells was determined by quantitative RT-PCR. (B) TNF-α production in the basolateral compartment was determined by a L929 cytotoxicity assay. The values represent the means ± SE. Experiments were repeated for three times in triplicate. *P<0.05, **P<0.01 vs. LPS control.

We hypothesized that down-regulation of the cell surface TNFR1 by lentinan treatment may occur via receptor endocytosis. It has been reported that endocytosis of TNF receptor superfamily is dependent on actin or clathrin polymerization [34]. We therefore utilized inhibitors to target these specific pathways and evaluate their relative contributions to lentinan inhibition of IL-8 mRNA expression in Caco-2. As shown in Fig. 5A, cytochalasin D, an antagonist of actin polymerization, did not affect lentinan inhibition of IL-8 mRNA expression in Caco-2 cells. In contrast, monodansylcadaverine, a hydrophobic amine which inhibits clathrin-dependent endocytosis by affecting the function of clathrin and clathrin-coated vesicles [35], [36], canceled lentinan inhibition of IL-8 mRNA expression to LPS control level. However, TNF-α production from LPS-stimulated RAW264.7 cells was not affected by these endocytosis inhibitors compared to non-treatment LPS control (Fig. 5B). These results indicate that lentinan stimulation induce a clathrin-mediated endocytosis of TNFR1 in Caco-2 cells, resulting in inhibition of IL-8 mRNA expression.

### Inhibitory Effect of Lentinan on TNFR1 Expression in Caco-2 Cells

**Figure 6:**
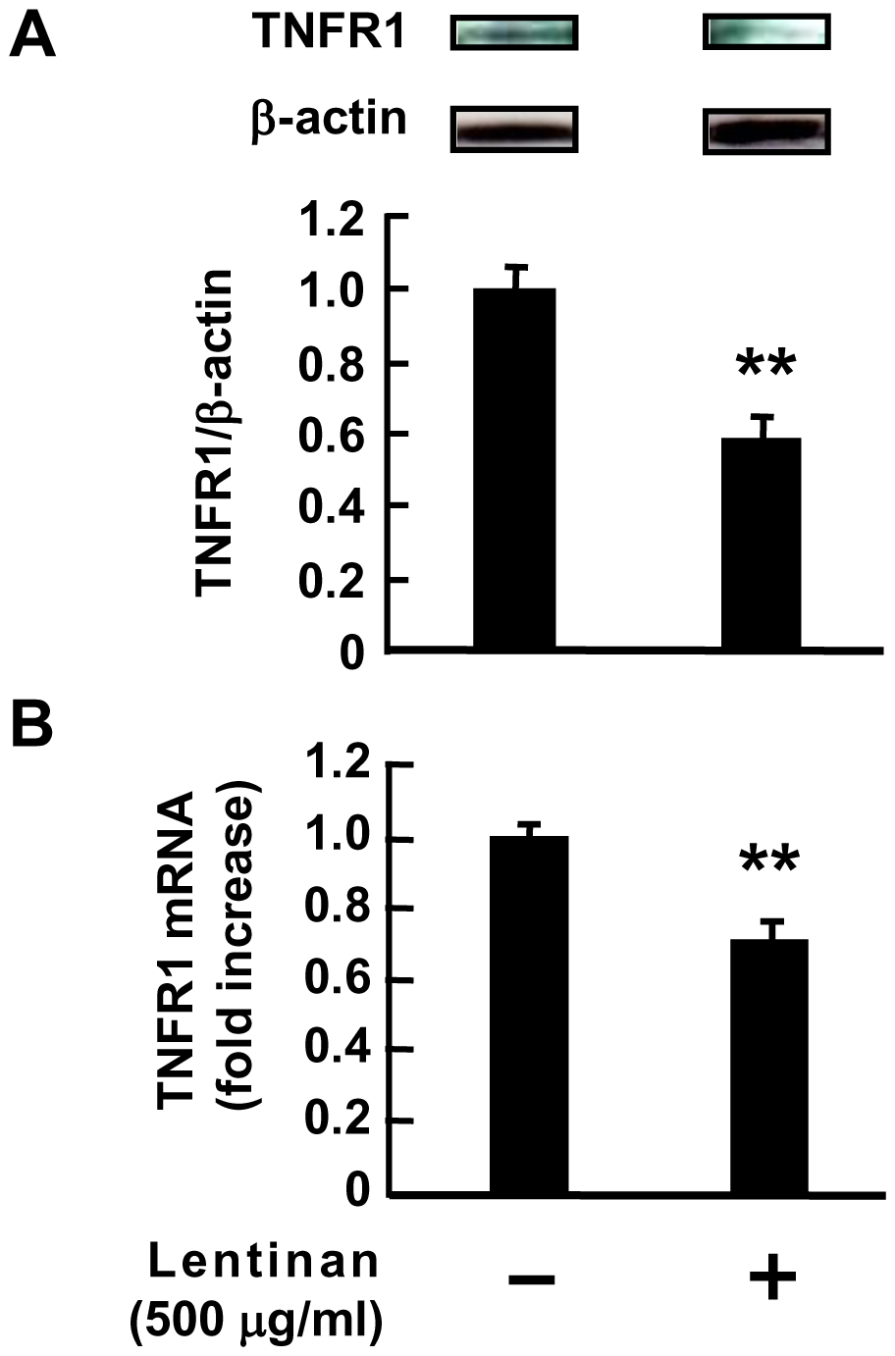
Lentinan inhibits TNFR1 protein and mRNA expression in Caco-2 cells. (A) Lentinan (500 µg/ml) was added into the apical compartment of Caco-2/RAW264.7 co-culture model for 3 h. Subsequently, LPS was added to the basolateral compartment at a concentration of 1 ng/ml, followed by incubation for an additional 2 h. Then, Western blot analysis of TNFR1 was performed on total cell extracts from Caco-2 cells. (B) Lentinan (500 µg/ml) was added into the apical compartment of Caco-2/RAW264.7 co-culture model for 2 h. The TNFR1 mRNA expression in Caco-2 cells was determined by quantitative RT-PCR. The values represent the means ± SE. Experiments were repeated for three times in triplicate. **P<0.01.

Next, we investigated the expression of TNFR1 in Caco-2 cells after lentinan-induced endocytosis. As shown in Fig. 6A, western blot analysis revealed that the level of TNFR1 protein in whole cell extracts from Caco-2 cells was significantly decreased to approximately 60% of its control level by lentinan (500 µg/ml) treatment (*P*<0.01) for 5 h. Moreover, quantitative RT-PCR analysis revealed that 2 h of lentinan treatment also suppressed TNFR1 mRNA expression in Caco-2 cells (*P*<0.01). The inhibition rate reached approximately 30% (Fig. 6B). In this model, Caco-2 cells only expressed IL-8 when the cells were stimulated by rmTNF-α from basolateral side (Fig. S1). We hypothesized that the ability of lentinan to decrease TNFR1 expression in Caco-2 cells involves alteration of the receptor distribution. To determine the distribution of TNFR1 in Caco-2 cells, immunofluorescent analysis was performed. As expected, z-stack image scanning revealed that TNFR1 on the basolateral side of the cells was remarkably decreased by lentinan treatment while it was uniformly distributed from apical to basolateral side in the absence of lentinan (Fig. S3). These results indicate that lentinan exerts a suppressive effect on TNFR1 expression after inducing the receptor endocytosis, resulting in the absence of TNFR1 on the basolateral side of Caco-2 cells.

### Inhibitory Effect of Lentinan on TNFR1 mRNA Expression *in vivo*

**Figure 7:**
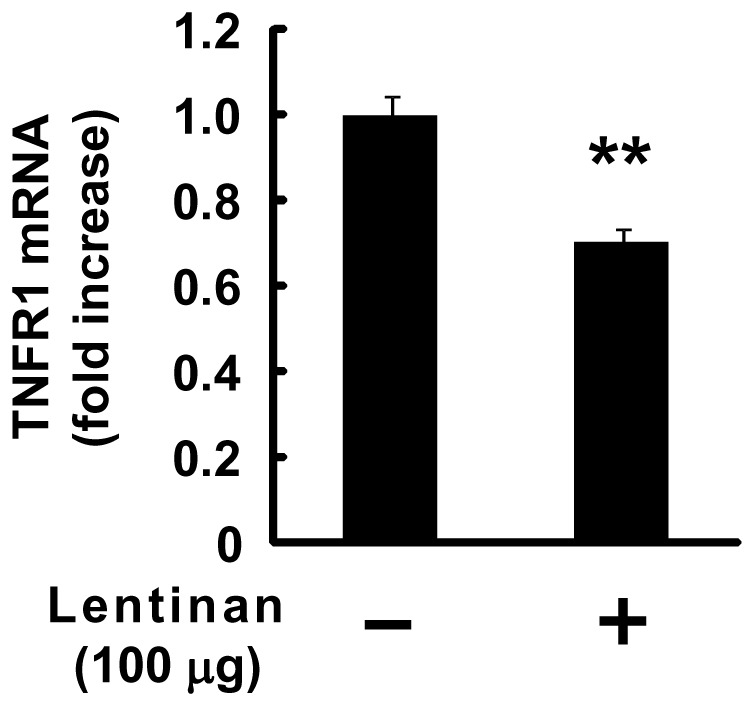
Lentinan suppresses TNFR1 mRNA expression in IECs ***in vivo*** **.** One hundred microliter of lentinan solution (1 mg/ml) or vehicle was injected into the separated two ligated intestinal loops which were located in mouse ileum, respectively. After incubation for 1 h, the mice were killed and the ligated intestinal loops were excised from the intestine. IECs were isolated and TNFR1 mRNA expression in the cells was determined by quantitative RT-PCR. The values represent the means ± SE. Experiments were repeated for three times in triplicate. **P<0.01 vs. vehicle control.

To investigate the effect of lentinan on TNFR1 mRNA expression *in vivo*, intestinal ligated loop assay was performed. As shown in Fig. 7, lentinan treatment significantly decreased TNFR1 mRNA expression in mouse IECs compared to vehicle control (*P*<0.01). The inhibition rate reached approximately 30% (Fig. 7). This result suggests that modulation of TNFR1 expression in IECs may be involved in gut anti-inflammatory activity of lentinan *in vivo*.

### Effect of Anti-lentinan Polyclonal Ab on Lentinan Inhibition of IL-8 mRNA Expression in Caco-2 Cells

**Figure 8:**
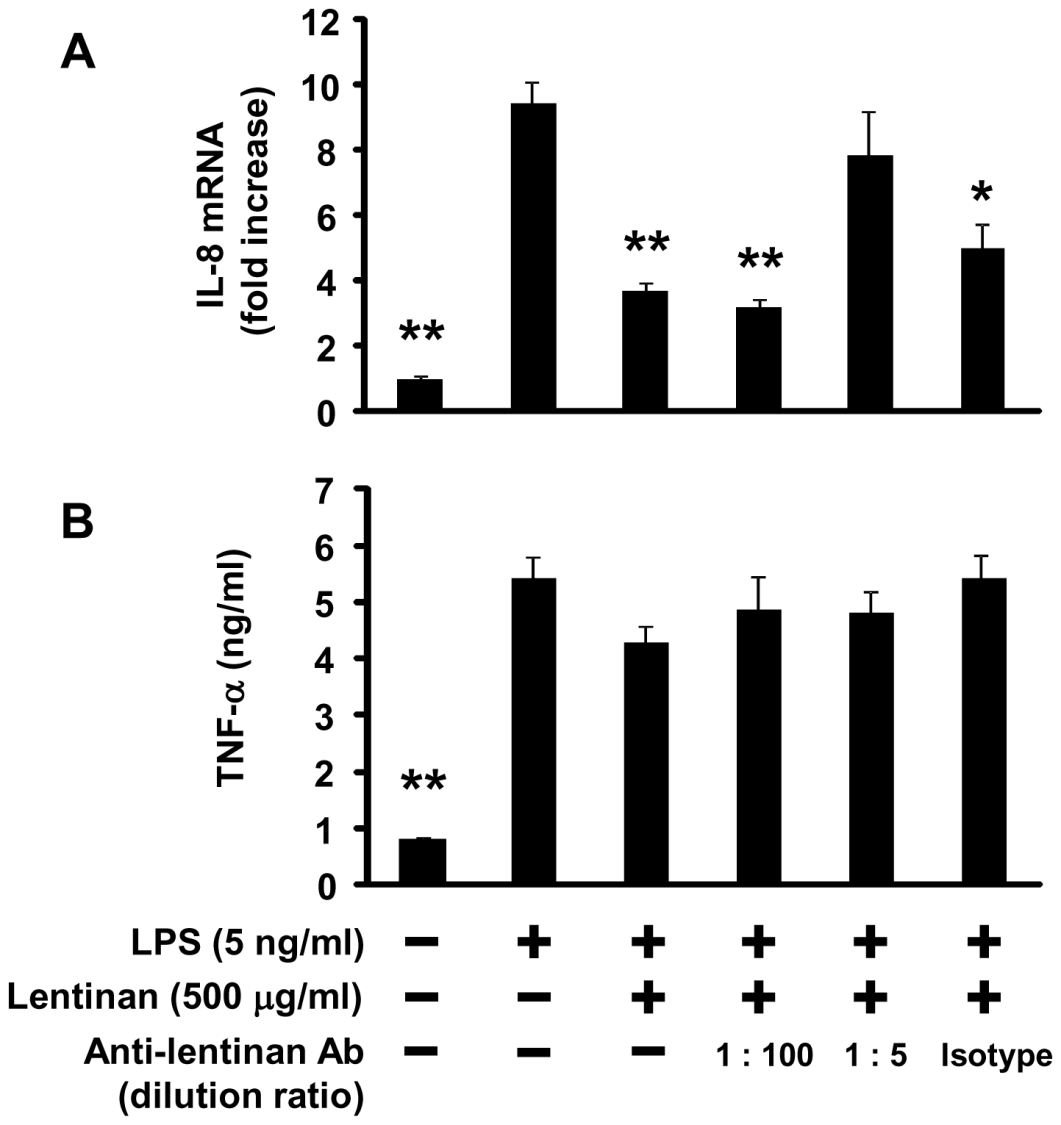
Effect of anti-lentinan polyclonal Ab on lentinan inhibition of IL-8 mRNA expression in Caco-2 cells. A rabbit polyclonal anti-lentinan Ab was diluted with PBS at ratios of 1∶5 or 1∶100, and then mixed with lentinan solution and incubated on ice for 30 min. A rabbit polyclonal isotype control Ab was used as control at the same protein concentration as anti-lentinan Ab (dilution ratio of 1∶5). Antibody-treated lentinan (500 µg/ml) was added into the apical compartment of a co-culture model for 3 h. Subsequently, LPS was added to the basolateral compartment at a concentration of 5 ng/ml, followed by incubation for an additional 3 h. IL-8 mRNA expression in Caco-2 cells was determined by quantitative RT-PCR. (B) TNF-α production in the basolateral compartment was determined by a L929 cytotoxicity assay. The values represent the means ± SE. Experiments were repeated for three times in triplicate. *P<0.05, **P<0.01 vs. LPS control.

Although we showed that lentinan exhibits intestinal anti-inflammatory activity by inducing TNFR1 endocytosis in IECs, it remains unclear how these cells recognize lentinan. In order to assess the mechanism of how Caco-2 cells recognize lentinan, the effect of anti-lentinan rabbit polyclonal Ab on lentinan inhibition of IL-8 mRNA expression in Caco-2 cells was investigated. Treatment of anti-lentinan Ab did not have a significant effect on TNF-α production from RAW264.7 cells in an *in vitro* gut inflammation model (Fig. 8B). However, treatment of anti-lentinan Ab at a dilution ratio of 1∶5, but not isotype control Ab, canceled lentinan inhibition of IL-8 mRNA expression in Caco-2 cells (Fig. 8A). These results suggest that Caco-2 cells may recognize the structure of lentinan via the cell surface receptor, followed by the subsequent TNFR1 endocytosis.

## Discussion

The mainstream treatments used to manage IBD are largely based on immunosuppressive approaches with broad acting agents such as prednisone, cyclosporin A, and tacrolimus [37]. Although they are relatively effective, a number of patients develop significant side effects and/or become unresponsive to them. The perception that alternative medicine is healthier than classical therapeutic options, have led a growing segment of the population to seek alternative treatments to ameliorate various disorders including chronic intestinal inflammation [38]. However, the absence of empirical data showing their efficacy and mechanisms of action prevents their incorporation into mainstream medicine. Meanwhile, it has been reported that mushroom-derived β-glucan exhibits immune activating properties [11]. Although it has been reported that the yeast zymosan induces immunological tolerance and regulatory antigen presenting cells into secreting abundant IL-10 but little or no IL-6 or IL-12 p70 [39], it is unknown whether mushroom-derived β-glucan can also induce immunosuppressive effects such as anti-inflammatory effects. In the present study, we investigated whether lentinan, a dietary β-1,3;1,6-glucan derived from *Lentinula edodes*, exerts anti-inflammatory activities using an *in vivo* and an *in vitro* model of gut inflammation, and we provide evidences that lentinan inhibits gut inflammation through modulation of TNFR1 expression in IECs.

Plant polysaccharides have been previously shown to reduce the extensive colonic damage in experimental colitis [40]–[42], but little is known about the effect of supplementing edible mushroom glucans in intestinal inflammation. Lentinan significantly improved body weight loss, shortening of colon length, and histological scores which were used to assess the degree of gut inflammation. We also showed that lentinan treatment in DSS mice attenuated the increase in IL-1β and IFN-γ significantly in colon segments. Pro-inflammatory cytokines are known to play an important role in inflammation of the intestinal mucosa [43]. Specifically, increased levels of TNF-α, IL-1β, IFN-γ, IL-6, and IL-8 have been reported in ulcerative colitis patients [44], [45]. IL-1β is a key cytokine involved in up-regulating the production of TNF-α, IL-6, and IL-8 [46], resulting in injury of intestinal epithelial tight junction barrier via up-regulating the production of myosin L chain kinase (MLCK) [47]. These results suggest that oral administration of lentinan exhibits anti-inflammatory activities in DSS-induced colitis mice through inhibition of pro-inflammatory cytokines production. Furthermore, in order to unveil the mechanism of intestinal anti-inflammatory activity of lentinan exhibited *in vivo*, we used a gut inflammatory model with co-culture system as described in our previous study.

Lentinan suppressed IL-8 gene expression without affecting TNF-α production. Since lentinan was not detected in the basolateral compartment of this gut inflammation model (data not shown), it was ascertained that lentinan could not penetrate the Caco-2 monolayer. These results indicate that the inhibitory effect of lentinan on IL-8 mRNA expression is acted through the interaction between lentinan and Caco-2 cells. It has been reported that the transcriptional regulation of IL-8 genes is associated with the activation of such nuclear transcription factors as NF-κB, activator protein (AP)-1, and CCAAT/enhancer binding protein (C/EBP) [30], [48], [49]. Among these, the NF-κB binding site on genes has been found to be a functionally important regulatory element for IL-8 gene expression in human epithelial cells [50]. NF-κB is a complex composed of p65 and p50 and translocates to the nucleus in the presence of stimulations such as TNF-α and IL-1β [50]. As expected, the increase in the NF-κB p65 level in the nucleus was observed in this Caco-2/RAW264.7 gut inflammation model. Treatment of lentinan suppressed the increase of NF-κB level in the nucleus of Caco-2 cells. Since TNFR1 mediates TNF-α induced NF-κB activation [31], [32] and is crucial for murine colitis induction [33], subsequently we investigated the effect of lentinan on the receptor.

Upon ligand binding, TNFR1 initiates intracellular signal transduction. Two signaling pathways, leading to anti-apoptotic or pro-apoptotic responses, are known. First, ligand-activated TNFR1 promotes the activation of the transcription factor NF-κB via recruitment of TNFR-associated death domain (TRADD) protein, receptor-interacting protein-1 (RIP1) and TNFR-associated protein-2 (TRAF2) at the cell surface, leading to anti-apoptotic and pro-inflammatory responses [34]. Second, TNFR1 bound with ligand is internalized via clathrin-dependent endocytosis and TRADD recruits FAS-associated death domain protein (FADD) and caspase-8 to the internalized receptosomes, leading to cytotoxic and pro-apoptotic responses [34]. In this study, flow cytometric analysis revealed that surface levels of TNFR1 in Caco-2 cells were decreased by lentinan treatment. In addition, TNF-α-induced reduction of transepithelial electrical resistance (TER) of Caco-2 cell monolayer was not observed in lentinan-treated cells for 48 h though a significant reduction was observed in vehicle-treated cells (data not shown). It was reported that endocytosis of epidermal growth factor receptor (EGFR) in mouse colon epithelial cells resulted in desensitization because of reducing receptor accessible to its ligand [51]. These evidences indicate that TNFR1 endocytosis in Caco-2 cells by lentinan stimulation may lead to inhibit IL-8 mRNA expression via reduction of cell surface TNFR1 without inducing apoptosis. Since monodansylcadaverine altered lentinan inhibition of IL-8 mRNA expression in Caco-2 cells, it is thought that clathrin-mediated endocytosis may be involved in the pathway.

Chin *et al*. demonstrated that the receptor internalization and degradation (RID) complex, composed of two RIDα and one RIDβ protein subunits, of adenovirus plays an important role in modulating the immune response by down-regulating the surface levels of TNFR1, thereby inhibiting NF-κB activation [52]. They showed that RID is able to associate with TNFR1 on the cell surface, both RID and clathrin play an important role in mediating delivery of TNFR1 to intracellular sites that accelerate its degradation [53]. In this study, the treatment of lentinan exerted an inhibitory effect on epithelial TNFR1 expression in protein and mRNA level. In addition, lentinan also inhibited TNFR1 mRNA expression in IECs *in vivo*. These results suggest that the suppressive effect of lentinan on epithelial TNFR1 expression after the receptor endocytosis may be one of the key mechanisms for its anti-inflammatory activity on IECs. In order to obscure structure of lentinan, anti-lentinan polyclonal Ab was used in this study. As a result, treatment of anti-lentinan Ab canceled lentinan inhibition of IL-8 mRNA expression in Caco-2 cells. Although the direct interaction between lentinan and TNFR1 remains unclear, this evidence indicates that the structure of lentinan may be important for its effect on IECs. Further studies about the association between lentinan and TNFR1 or other cell surface receptors such as Dectin-1 which is known to recognize β-glucan are necessary to fully understand the mechanism of anti-inflammatory activity of lentinan.

In summary, a putative mechanism for the suppressive effect of lentinan on IL-8 mRNA expression in Caco-2 cells was demonstrated, as follows: 1) lentinan treatment induced TNFR1 endocytosis and exerted a suppressive effect on the TNFR1 expression of Caco-2 cells; 2) NF-κB translocation into the nucleus was decreased; 3) the increase in IL-8 mRNA expression was suppressed. Finally, elucidating the entire mechanism of the suppressive effect on intestinal inflammation induced by dietary β-1,3;1,6-glucan will provide valuable information toward the establishment of a new β-glucan therapy for treating patients with IBD.

## Supporting Information

Figure S1 **TNF-α stimulation from basolateral side is necessary for IL-8 mRNA expression in Caco-2 cells.** (A) Caco-2 cells were treated with rmTNF-α (100 ng/ml) from the apical or basolateral side for 3 h. IL-8 mRNA expression in Caco-2 cells was detected by quantitative RT-PCR. **P<0.01 vs. control. (B) Caco-2 cells were grown as monolayers in the upper chamber (normal) or on the underside of the transwell inserts (inverted) to form stable, polarized monolayers. The transwell inserts were added into multiple plate wells preloaded with RAW264.7 cells, and incubated for 3 h. Then, LPS was added into the lower chamber, followed by additional incubation for 3 h. IL-8 mRNA expression in Caco-2 cells was detected by quantitative RT-PCR. (C) TNF-α production in the basolateral compartment was determined by a L929 cytotoxicity assay. Black columns indicate normal and gray columns indicate inverted. The values represent the means ± SE (*n = *3). **P<0.01.
(PPTX)

Figure S2 **NF-κB p65 protein nuclear translocation in Caco-2 cells.** (A) Caco-2 cells were incubated with RAW264.7 cells for 3 h. Subsequently, LPS was added to the basolateral compartment up to a final concentration of 10 ng/ml, followed by incubation for an additional 3 h. Western blot analysis of the NF-κB p65 subunit was performed on nuclear extracts from Caco-2 cells incubated for various times. (B) TNF-α production in the basolateral compartment was determined by a L929 cytotoxicity assay. The values represent the means ± SE (*n* = 3).
(PPTX)

Figure S3 **Lentinan induces alteration of TNFR1 distribution in Caco-2 cells.** Lentinan (500 µg/ml) or vehicle was added into the apical compartment of Caco-2/RAW264.7 co-culture model for 5 h at 37°C. Then, immunofluorescent analysis of TNFR1 in Caco-2 cells was performed. Z-stack images of sample-treated Caco-2 cell monolayers were obtained by using a confocal laser scanning microscope. Immunofluorescent staining of TNFR1 (green) in Caco-2 cells, costained with phalloidin (red) for F-actin and TO-PRO-3 iodide (blue) for nuclei.
(PPTX)

Text S1 **Lentinan content measurement.**
(DOC)

Text S2 **Immunofluorescence staining of TNFR1 in Caco-2 cells.**
(DOC)
