# Supplementary figures and images for: Intestinal Anti-Inflammatory Activity of Lentinan: Influence on IL-8 and TNFR1 Expression in Intestinal Epithelial Cells
Source: PLoS One. 2013 Apr 22;8(4):e62441. doi: 10.1371/journal.pone.0062441 (PMC3632531; doi:10.1371/journal.pone.0062441)

## Slide 1
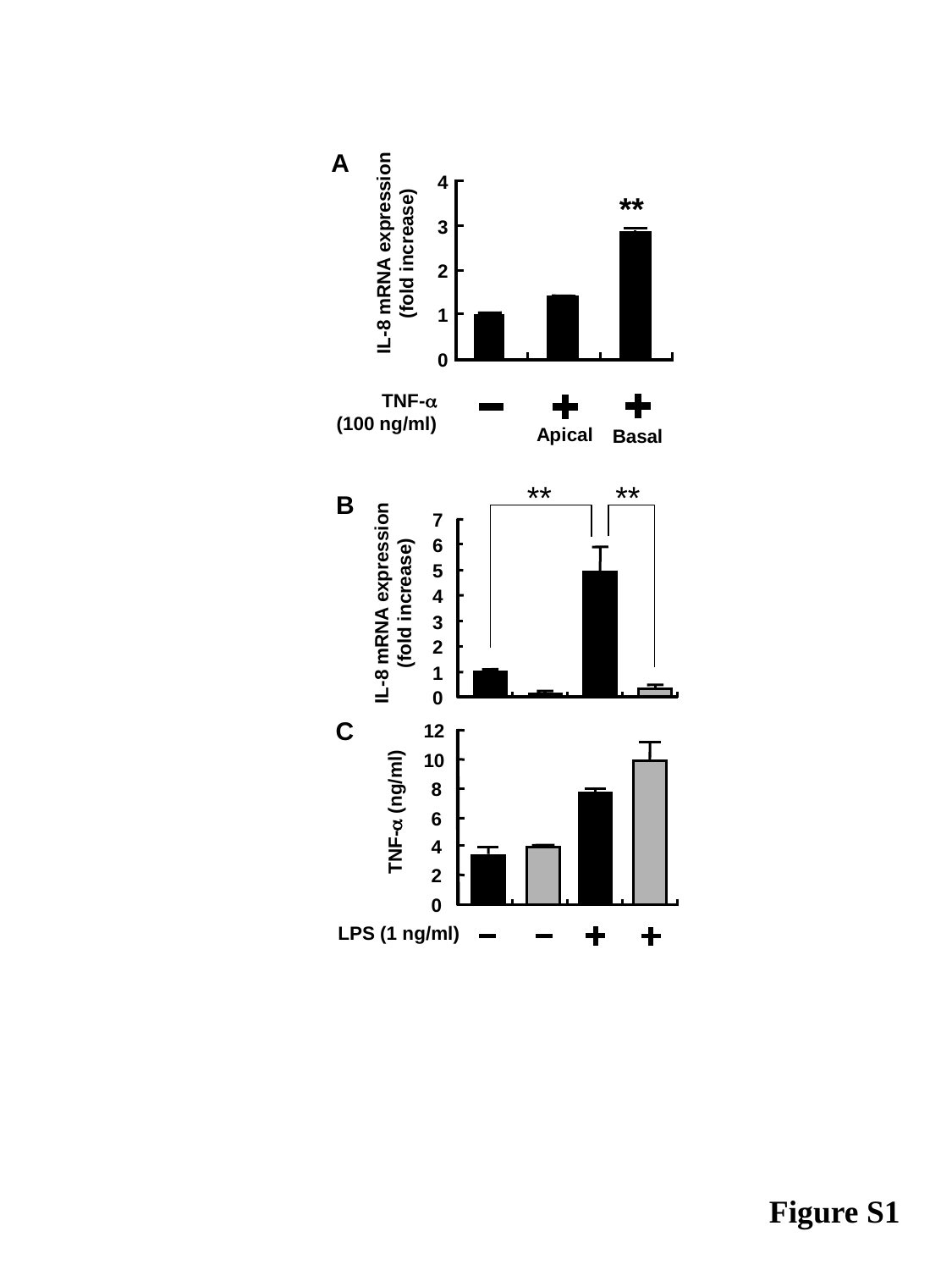

Figure S1

Supplement: Figure S1 — TNF-α stimulation from basolateral side is necessary for IL-8 mRNA expression in Caco-2 cells. (A) Caco-2 cells were treated with rmTNF-α (100 ng/ml) from the apical or basolateral side for 3 h. IL-8 mRNA expression in Caco-2 cells was detected by quantitative RT-PCR. **P<0.01 vs. control. (B) Caco-2 cells were grown as monolayers in the upper chamber (normal) or on the underside of the transwell inserts (inverted) to form stable, polarized monolayers. The transwell inserts were added into multiple plate wells preloaded with RAW264.7 cells, and incubated for 3 h. Then, LPS was added into the lower chamber, followed by additional incubation for 3 h. IL-8 mRNA expression in Caco-2 cells was detected by quantitative RT-PCR. (C) TNF-α production in the basolateral compartment was determined by a L929 cytotoxicity assay. Black columns indicate normal and gray columns indicate inverted. The values represent the means ± SE (n = 3). **P<0.01. (PPTX) [file pone.0062441.s001.pptx]

## Slide 1
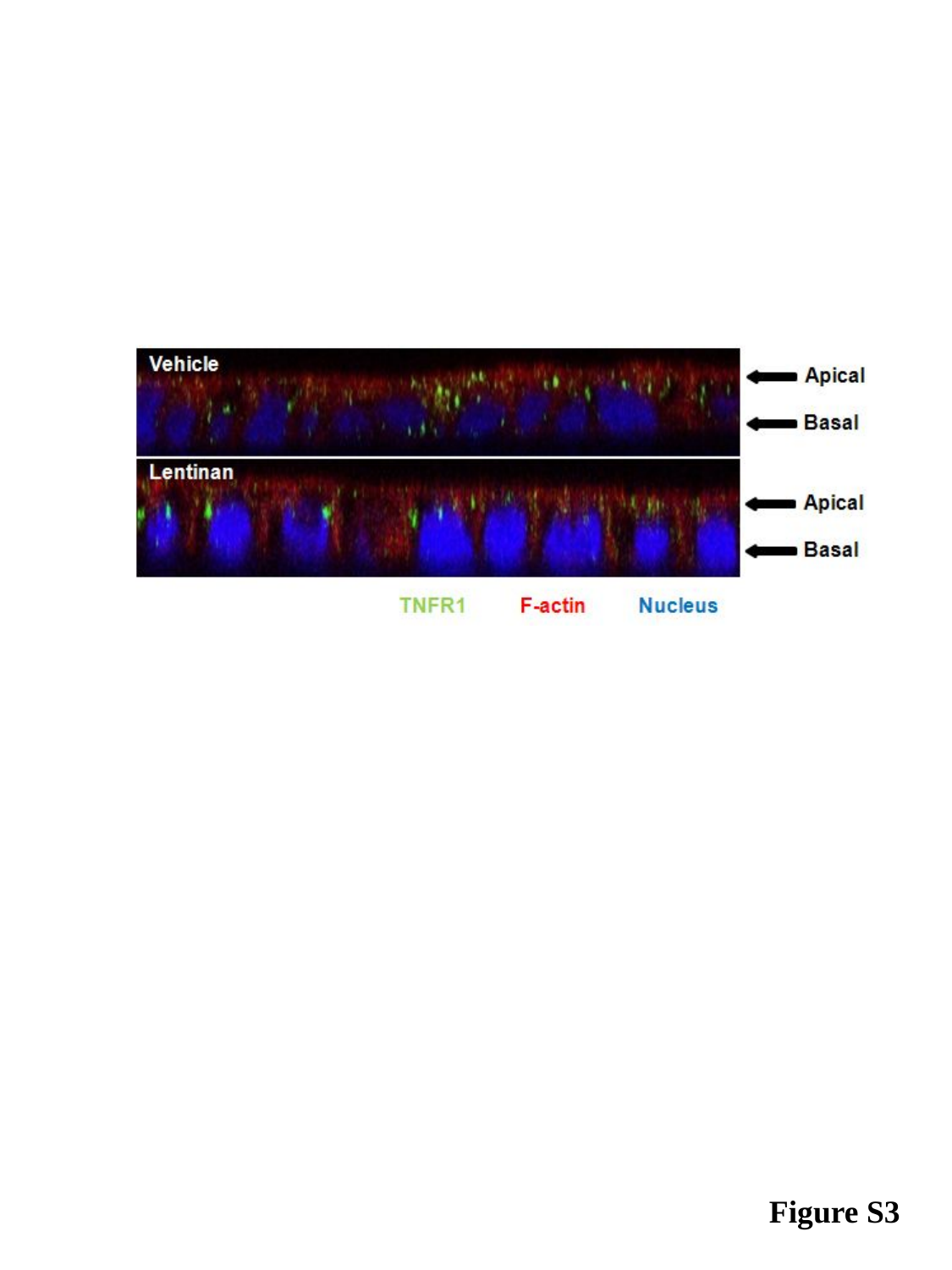

Figure S3

Supplement: Figure S3 — Lentinan induces alteration of TNFR1 distribution in Caco-2 cells. Lentinan (500 µg/ml) or vehicle was added into the apical compartment of Caco-2/RAW264.7 co-culture model for 5 h at 37°C. Then, immunofluorescent analysis of TNFR1 in Caco-2 cells was performed. Z-stack images of sample-treated Caco-2 cell monolayers were obtained by using a confocal laser scanning microscope. Immunofluorescent staining of TNFR1 (green) in Caco-2 cells, costained with phalloidin (red) for F-actin and TO-PRO-3 iodide (blue) for nuclei. (PPTX) [file pone.0062441.s003.pptx]
