# Supplementary material for: Intestinal Anti-Inflammatory Activity of Lentinan: Influence on IL-8 and TNFR1 Expression in Intestinal Epithelial Cells
Source: PLoS One. 2013 Apr 22;8(4):e62441. doi: 10.1371/journal.pone.0062441 (PMC3632531; doi:10.1371/journal.pone.0062441)
Supplement: Figure S2 — NF-κB p65 protein nuclear translocation in Caco-2 cells. (A) Caco-2 cells were incubated with RAW264.7 cells for 3 h. Subsequently, LPS was added to the basolateral compartment up to a final concentration of 10 ng/ml, followed by incubation for an additional 3 h. Western blot analysis of the NF-κB p65 subunit was performed on nuclear extracts from Caco-2 cells incubated for various times. (B) TNF-α production in the basolateral compartment was determined by a L929 cytotoxicity assay. The values represent the means ± SE (n = 3). (PPTX) [file pone.0062441.s002.pptx]

## Slide 1
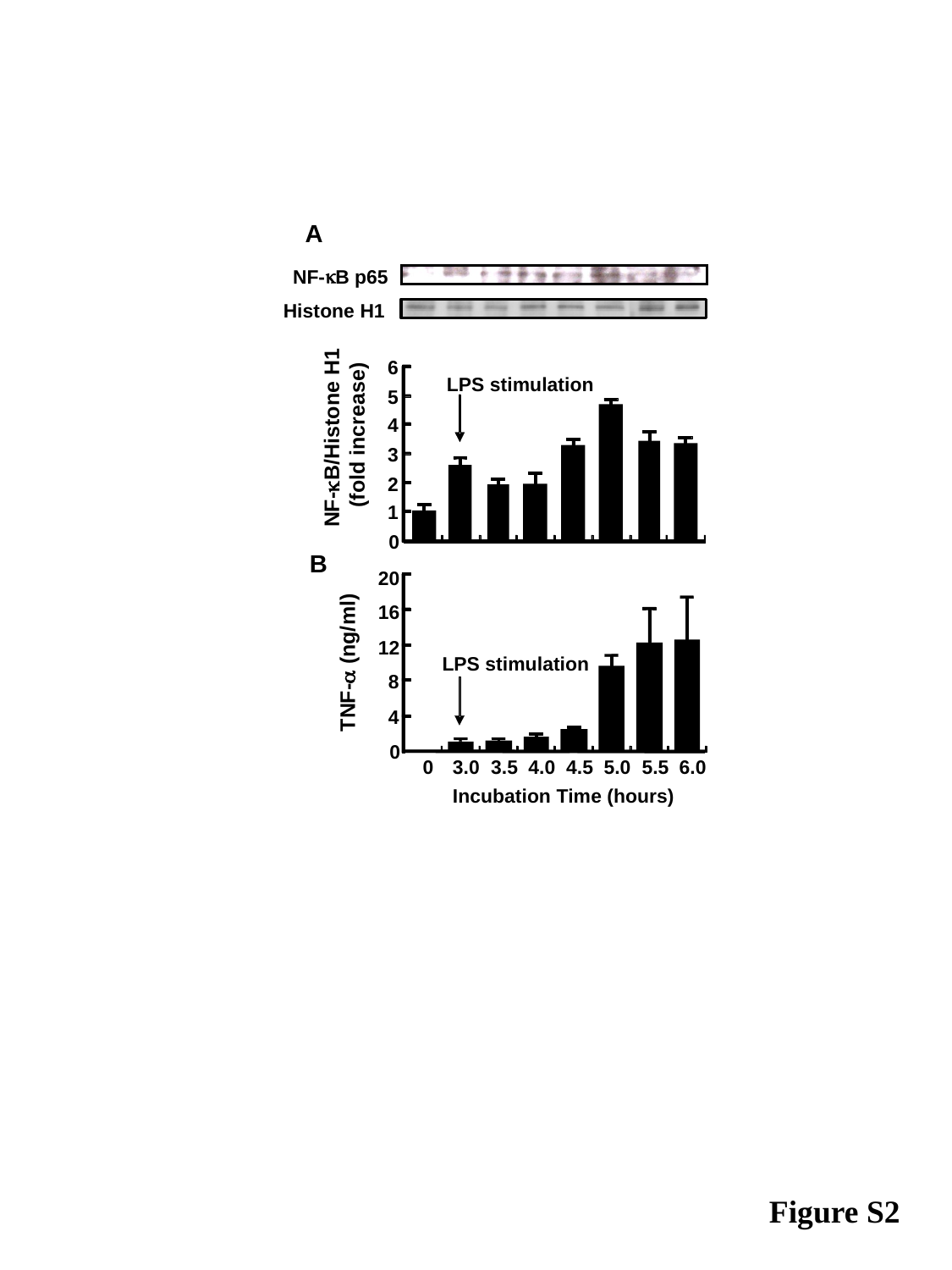

A
k
NF
-
B p65
Histone H1
6
LPS stimulation
5
NF-κB/Histone H1
 (fold increase)
4
3
2
1
0
B
20
16
12
TNF-α (ng/ml)
LPS stimulation
8
4
0
0
3.0
3.5
4.0
4.5
5.0
5.5
6.0
Incubation Time (hours)
Figure S2
