# Supplementary material for: Intestinal Anti-Inflammatory Activity of Lentinan: Influence on IL-8 and TNFR1 Expression in Intestinal Epithelial Cells
Source: PLoS One. 2013 Apr 22;8(4):e62441. doi: 10.1371/journal.pone.0062441 (PMC3632531; doi:10.1371/journal.pone.0062441)
Supplement: Text S1 — Lentinan content measurement. (DOC) [file pone.0062441.s004.doc]

**Supporting Information**

Supplementary Materials and Methods

***Lentinan content measurement***

For measuring lentinan contents, the culture supernatants from the apical and basolateral sides of co-culture system were collected. The lentinan contents of the culture supernatants were quantified using the enzyme-linked immunosorbent assay (ELISA) inhibition technique with anti-lentinan polyclonal Ab which was previously reported by Mizuno *et al*., [19].
