# Supplementary material for: Intestinal Anti-Inflammatory Activity of Lentinan: Influence on IL-8 and TNFR1 Expression in Intestinal Epithelial Cells
Source: PLoS One. 2013 Apr 22;8(4):e62441. doi: 10.1371/journal.pone.0062441 (PMC3632531; doi:10.1371/journal.pone.0062441)
Supplement: Text S2 — Immunofluorescence staining of TNFR1 in Caco-2 cells. (DOC) [file pone.0062441.s005.doc]

**Supporting Information**

Supplementary Materials and Methods

***Immunofluorescence staining*** ***of TNFR1 in Caco-2 cells***

After the co-culture experiments, Caco-2 cells on the transwell membrane were fixed with 4% paraformaldehyde in PBS for 15 min at 4 °C, and washed with PBS for three times at room temperature. The membrane was blocked with 2% bovine serum albumin in PBS containing 0.1% Triton X-100 for 30 min at room temperature, and washed with PBS containing 0.1% Triton X-100 for three times. The cells were incubated with a 1:50 dilution of anti-human TNFR1 antibody (mouse monoclonal IgG) at room temperature for 2 h. The cells were stained with Alexa Fluor488-conjugated anti-mouse IgG (H + L) goat antibody as secondary antibodies (1:400 dilution; Molecular Probes, Eugene, OR) for 1 h at room temperature. Nucleic acids were stained with TO-PRO-3 iodide (1:1,000; Molecular Probes), and F-actin was stained with Alexa Fluor546-conjugated phalloidin (1:50; Molecular Probes). Images were acquired using a fluorescence microscope (IX71; Olympus, Tokyo, Japan). TNFR1 staining appeared green and nuclei staining blue, and F-actin staining red.
